# The Effect of Weight Loss on Serum Mannose-Binding Lectin Levels

**DOI:** 10.1155/2012/354894

**Published:** 2012-11-14

**Authors:** P. H. Høyem, J. M. Bruun, S. B. Pedersen, S. Thiel, B. Richelsen, J. S. Christiansen, T. K. Hansen

**Affiliations:** ^1^Department of Endocrinology and Internal Medicine MEA, Aarhus University Hospital, 8000 Aarhus, Denmark; ^2^Institute of Medical Microbiology and Immunology, Aarhus University, 8000 Aarhus, Denmark

## Abstract

*Background*. Serum levels of the mannose-binding lectin (MBL), which is an activator of the complement system, have been considered as a pathogenic factor in a broad range of diseases, and means of modulating MBL are therefore being evaluated. In this study we examine the effects of weight loss on MBL levels, and in continuation of this if MBL is synthesized in human adipose tissue. *Methods*. 36 nondiabetic obese subjects received a very low-calorie diet (VLCD) of 800 kcal/day for 8 weeks. Blood samples were collected at baseline and after VLCD. Furthermore, we measured MBL mRNA levels by the real-time RT-PCR on human adipose tissue compared to liver tissue. *Results*. The mean body weight was reduced from 106.3 ± 2.6 kg to 92.8 ± 2.4 kg, *P* < 0.0001. Median MBL at baseline was 746 **μ**g/L (IQR 316–1190) versus 892 **μ**g/L (IQR 336–1511) after 8 weeks, *P* = 0.23. No correlations were found between weight loss and changes in MBL (*r* = -0.098, *P* = 0.57). MBL real-time RT-PCR showed no expression of mRNA in adipose tissue, but as expected a good expression in liver tissue was seen. *Conclusions*. MBL levels are not affected by weight loss and MBL is not synthesized in human adipose tissue.

## 1. Introduction

Mannose-binding lectin (MBL) is a serum protein known to be synthesized by hepatocytes. 

MBL exerts an important role in the innate immune system, where it upon binding to carbohydrate patterns of microorganisms activates the lectin pathway of the complement system through MBL-associated serine protease 1, 2, and 3 [1, 2]. This leads to recruitment of inflammatory cells, opsonization, and formation of the membrane attack complex [3], which causes destruction of the microorganisms. 

Serum MBL level is dependent on genotype and, because of commonly occurring polymorphisms in the MBL encoding gene *MBL2*, the interindividually expressed serum level of MBL ranges from a few *μ*g/L to several thousand *μ*g/L. In healthy subjects approximately 56% have a high MBL encoding genotype (homozygote wild type), 40% have an intermediate MBL encoding genotype (mutation in one allele), and 4% have a low MBL encoding genotype (mutations in both alleles), the latter being functionally deficient in MBL [4], the most common immunodeficiency described.

Several clinical studies have shown low MBL levels to be associated with increased susceptibility or to poor outcome of infections. This lack of defense against invading microorganisms may be important especially in certain situations, for example, in infants with an immature immune system [5–7] and in adults with a compromised immune system [8, 9], as the immune system normally features a degree of redundancy.

Under disease circumstances other than an infection, activation of the complement system can be adverse and low MBL level may here confer an advantage. Keller et al. found that healthy men with low MBL levels are protected from cardiovascular disease [10].

Normally, MBL does not bind to the body's own cells, but may react with altered self-structures, for example, cells exposed to ischaemia/reperfusion injury [11, 12] or to apoptotic cells [13]. Also in autoimmune disease, as rheumatoid arthritis, MBL might bind to altered self-structures [14]. 

In a prospective study with 15 years of followup, our group documented an increased mortality among type 2 diabetic patients with high MBL levels compared with those with low MBL levels [15]. Furthermore, patients with a history of cardiovascular disease had significantly higher MBL levels than patients without prior cardiovascular disease [15]. Moreover the association between a history of cardiovascular disease and high MBL levels has been shown in type 1 diabetic patients and also that patients with nephropathy had significantly higher MBL levels than patients with normoalbuminuria [16]. A plausible explanation for the association between high MBL levels and diabetic vascular complications could be MBL binding to glucosylated cells in the endothelium and promoting low-grade inflammation through complement activation, resulting in accelerated atherosclerosis.

Because of MBL's possible pathogenic involvement in a broad range of diseases it might comprise a potential target of treatment. Studies of direct inhibition of human MBL in vitro [17] the rat MBL in vivo [12] and downstream regulation of complement activation with anti-C5 complement antibody in humans [18] are being conducted.

MBL level is, as mentioned above, mostly dependent on genotype and though it is known to be affected by hormonal stimuli [19] and is as such a modifiable parameter, intraindividual variation in MBL is small over time [20, 21]. 

So far the effects of weight loss and changes in insulin resistance on MBL levels have only been poorly investigated and with conflicting results. Regarding weight loss induced by bariatric surgery in obese subjects, both increased levels [22] and unchanged levels [23] have been reported. In the present study we aimed to investigate whether weight loss caused by dietary intervention influences MBL levels and in addition to investigate whether adipose tissue might be able to produce MBL.

## 2. Research Design and Methods

We investigated the effect of weight loss and change in insulin resistance on MBL levels in an intervention study including 36 healthy and nondiabetic obese subjects (18 women and 18 men). The subjects received a very low-calorie diet (VLCD) of ~800 kcal/day for 8 weeks. 

Anthropometric data was obtained at baseline and after the 8 weeks of VLCD. Body composition (fat mass) and the percentage of body fat (fat mass/weight × 100%) were estimated by bioelectrical impedance using a multifrequency bioimpedance spectroscopy analyzer (SGB3; UniQuest Limited, Brisbane, Australia) as described by Heitmann [24].

We obtained fasting blood samples from the antecubital vein at baseline and after the 8 weeks of VLCD. Serum was separated and frozen at −80°C until the time of the analysis.

HOMA-IR was calculated as the product of the fasting plasma insulin level (microU/mL) and the fasting plasma glucose level (mmol/L), divided by 22.5.

Furthermore, to investigate if MBL is synthesized in adipose tissue real-time RT-PCR for MBL mRNA expression was performed on human adipose tissue obtained from fat biopsies from a subset of the participants and compared to commercial available human liver RNA.

The study was conducted according to the Declaration of Helsinki and was approved by the local Ethical Committee. Study participants gave a written informed consent before entering the study.

## 3. Blood Sample Assays

Serum glucose was measured by standard methods at the Clinical Biochemical Department, Aarhus University Hospital.

Serum insulin was measured by AutoDELFIA Insulin kit B080-101 (Perkin Elmer, Turku, Finland).

Serum MBL levels were measured by an immunoassay, as described previously [25], using an in-house time-resolved immunoflurometric assay with a lower detection level of 10 *μ*g/L. In brief, microtiter wells were coated with monoclonal anti-MBL antibody followed by incubation with samples diluted 200-fold. After washing, monoclonal anti-MBL antibody labeled with europium was added, and after incubation and washing, the amount of bound, labeled antibody was assessed by time-resolved fluorometry. A number of control serum samples covering different MBL levels were included in all assays.

## 4. MBL Real-Time RT-PCR 

For determination of MBL mRNA expression, RNA was isolated from adipose tissue obtained from a subset of the participants using TRIzol reagent as previously described [26]. Isolated total liver RNA was obtained from Ambion (Life Technologies Ltd., 3 Fountain Drive, Inchinnan Business Park, Paisley PA4 9RF, UK).

Reverse transcription was performed using random hexamer primers as described by the manufacturer (GeneAmp RNA PCR Kit from Perkin Elmer Cetus, Norwalk, CT). Then, PCR-mastermix containing the specific primers and Taq DNA polymerase (HotStar Taq, Quiagen Inc., USA) was added. 

The following primers were designed using the primer analysis software Oligo version 6.64: MBL2: GCAAACAGAAATGGCACGTAT and AGAGGCCTGGAACTTGACA, product length 139 bp and *β*-actin: ACGGGGTCACCCACACTGTGC and CTAGAAGCATTTGCGGTGGACGATG, product length 658 bp. Real-time quantitation of a target gene to *β*-actin mRNA was performed with a SYBR-Green real-time PCR assay using an ICycler from Bio-Rad. The target gene and *β*-actin mRNA were amplified in separate tubes. The increase in fluorescence was measured in real time during the extension step. The threshold cycle (Ct) was calculated and the relative gene expression was calculated essentially as described in the User Bulletin number 2, 1997, from Perkin Elmer (Perkin Elmer Cetus, Norwalk, CT). Briefly, the target gene (X0)-to-*β*-actin (R0) ratio in each sample before amplification was calculated as X0/R0 = *k*x1/((2**deltaCt)); delta Ct is the difference between Ct-target and Ct-reference, and *k* is a constant, set to 1. All samples were amplified in duplicate. A similar setup was used for negative controls except that the reverse transcriptase was omitted and no PCR products were detected under these conditions.

## 5. Statistical Analysis

Values are presented as means ± SE, except for MBL, which was nonnormally distributed, and values are given as medians with interquartile ranges (IQR). 

Paired or unpaired *t*-test was used for normally distributed variables as appropriate. For nonnormally distributed variables, comparisons between groups were performed using the Wilcoxon signed ranks test or the Mann-Whitney *U*-test as appropriate. Pearson's correlation was used for normally distributed variables, whereas Spearman's correlation with two-tailed probability values was used to estimate the strength of association between nonnormally distributed variables.

Statistical significance was assumed for *P* < 0.05. IBM SPSS version 20 was used for all calculations.

## 6. Results 

The participants' mean age was 43 years (range from 24 to 62 years). Their characteristics at baseline and after 8 weeks of VLCD are presented in Table 1. They had a mean body weight of 106.3 ± 2.6 kg  (range from 76.1 to 142.7 kg) and a mean body mass index (BMI) of 34.2 ± 0.5 kg/m^2^  (range from 29.5 to 40.8 kg/m^2^).

**Figure 1:**
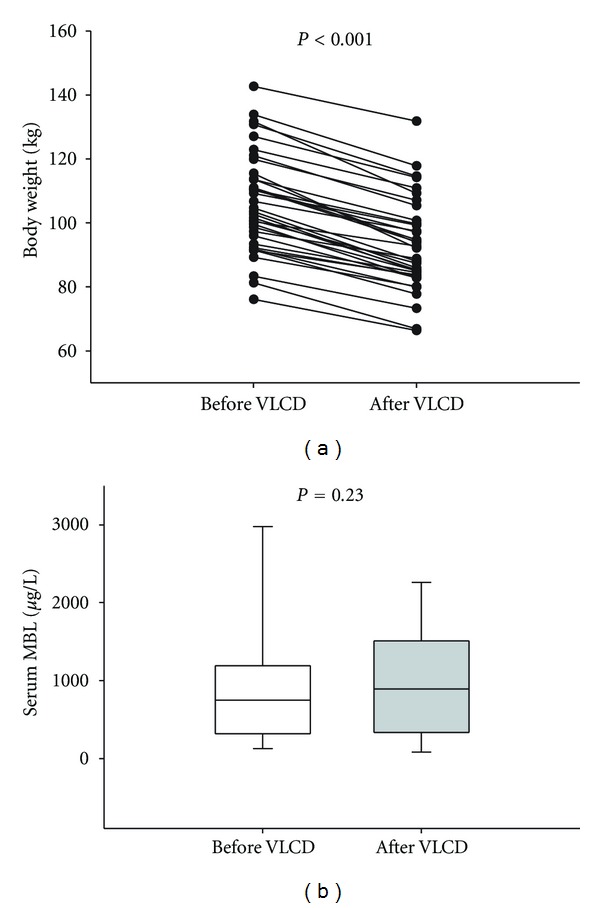
(a) Body weight before and after VLCD. (b) Serum MBL before and after VLCD.

After 8 weeks of VLCD the mean body weight was reduced by 12.7% (106.3 ± 2.6 kg versus 92.8 ± 2.4 kg, *P* < 0.001), Figure 1(a). Mean BMI was reduced by 12.6% (34.2 ± 0.5 kg/m^2^ versus 29.9 ± 0.6 kg/m^2^, *P* < 0.001) and the insulin resistance (HOMA-IR) was reduced by 50.2% (2.39 ± 0.32 versus 1.19 ± 0.22, *P* < 0.001). 

Measures of waist and fat mass (%) were also significantly reduced after 8 weeks. 

Median serum MBL levels did not change significantly during the 8 weeks of VLCD (746 (316–1190) *μ*g/L versus 892 (336–1511) *μ*g/L, *P* = 0.23), Figure 1(b).

Median serum MBL levels did not differ significantly between men and women at baseline nor after 8 weeks and the change in MBL levels remained insignificant when looking at men and women separately (data not shown).

MBL levels did not correlate with age (data not shown).

As expected, change in HOMA-IR correlated to change in weight (*r* = 0.595, *P* < 0.001).

Correlations between serum MBL at baseline and other baseline parameters are shown in Table 2. MBL at baseline was not correlated to baseline weight, BMI, or HOMA IR. Correlations between changes in serum MBL and changes in other parameters after the 8 weeks are shown in Table 2. No correlations were found between changes in MBL and weight loss, change in BMI, or change in HOMA-IR.

**Figure 2:**
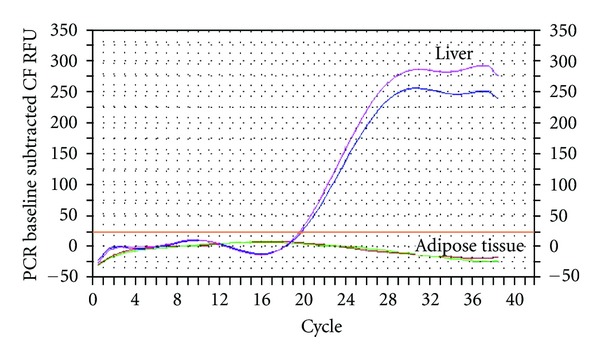
PCR of MBL in human liver and adipose tissue.

MBL real-time RT-PCR showed no expression of mRNA in adipose tissue but as expected a good expression in liver tissue, Figure 2.

## 7. Discussion

Our results suggest that serum MBL levels do not seem to be related to weight or insulin resistance. As expected, weight loss and reduction in insulin resistance were correlated. MBL levels were not affected by weight loss or changes in insulin resistance. 

The effect of weight loss on MBL levels has been poorly elucidated, and the reports so far have been conflicting. A small study of 10 severely obese, nondiabetic men and women found MBL levels to increase after weight loss obtained over 2 years (after biliopancreatic diversion). Furthermore, the study concluded that the change in MBL levels were positively associated with the increase in insulin sensitivity [22]. In contrast, another study from the same group found no significant change in MBL levels after weight loss (also after biliopancreatic diversion) in 10 normal glucose-tolerant obese women [23], data in support of our findings. 

We found no evidence that MBL is synthesized in the human adipose tissue. This further supports our finding of MBL not being related to weight loss.

The contradictory impacts of MBL under various conditions support a hypothesis of duality of MBL in a disease, and interventions, changing the MBL level, are currently being explored. Up to now, most of the focus has been made on antibodies to MBL or downstream complement factors, whereas the possible effect of lifestyle interventions remains sparsely explored.

## 8. Conclusion

We suggest that MBL levels may not be significantly modifiable by lifestyle interventions, such as weight loss, and that obesity and insulin resistance are not associated with MBL levels. MBL is not synthesized in human adipose tissue. Together, this indicates that decreased MBL levels are neither the result nor a contributing cause of obesity and insulin resistance. Rather interindividual differences in MBL depend primarily on the MBL genotype and constitute a preexisting condition, which might be an advantage or a disadvantage under different circumstances of health and disease.

## Figures and Tables

**Table 1:** Clinical characteristics of participants.

	Baseline	After 8 weeks of VLCD	*P* value
Weight (kg)	106.3 (±2.6)	92.8 (±2.4)	<0.001
BMI (kg/m^2^)	34.2 (±0.5)	29.9 (±0.6)	<0.001
Waist (cm)	112.8 (±1.8)	102.5 (±1.8)	<0.001
Fat mass (%)	37.0 (±1.4)	31.6 (±1.7)	<0.001
HOMA-IR	2.39 (±0.32)	1.19 (±0.22)	<0.001
Serum MBL (*μ*g/L) (median (IQR))	746 (316–1190)	892 (336–1511)	0.23

**Table:** (a)

Baseline	Baseline MBL
Weight	−0.022 (*P* = 0.899)
BMI	−0.013 (*P* = 0.942)
HOMA-IR	0.08(*P* = 0.964)

**Table:** (b)

Δ	ΔMBL
Weight	−0.098 (*P* = 0.571)
BMI	0.098 (*P* = 0.571)
HOMA-IR	−0.242 (*P* = 0.154)
